# Comparison of Resting-State Functional MRI Methods for Characterizing Brain Dynamics

**DOI:** 10.3389/fncir.2022.681544

**Published:** 2022-04-04

**Authors:** Eric Maltbie, Behnaz Yousefi, Xiaodi Zhang, Amrit Kashyap, Shella Keilholz

**Affiliations:** The Wallace H. Coulter Department of Biomedical Engineering, Georgia Institute of Technology and Emory University, Health Sciences Research Building, Atlanta, GA, United States

**Keywords:** dynamic functional connectivity, resting-state fMRI, k-means clustering, sliding window correlation, phase synchrony, co-activation patterns (CAPs), quasi-periodic pattern (QPP)

## Abstract

Resting-state functional MRI (fMRI) exhibits time-varying patterns of functional connectivity. Several different analysis approaches have been developed for examining these resting-state dynamics including sliding window connectivity (SWC), phase synchrony (PS), co-activation pattern (CAP), and quasi-periodic patterns (QPP). Each of these approaches can be used to generate patterns of activity or inter-areal coordination which vary across time. The individual frames can then be clustered to produce temporal groupings commonly referred to as “brain states.” Several recent publications have investigated brain state alterations in clinical populations, typically using a single method for quantifying frame-wise functional connectivity. This study directly compares the results of k-means clustering in conjunction with three of these resting-state dynamics methods (SWC, CAP, and PS) and quantifies the brain state dynamics across several metrics using high resolution data from the human connectome project. Additionally, these three dynamics methods are compared by examining how the brain state characterizations vary during the repeated sequences of brain states identified by a fourth dynamic analysis method, QPP. The results indicate that the SWC, PS, and CAP methods differ in the clusters and trajectories they produce. A clear illustration of these differences is given by how each one results in a very different clustering profile for the 24s sequences explicitly identified by the QPP algorithm. PS clustering is sensitive to QPPs with the mid-point of most QPP sequences grouped into the same single cluster. CAPs are also highly sensitive to QPPs, separating each phase of the QPP sequences into different sets of clusters. SWC (60s window) is less sensitive to QPPs. While the QPPs are slightly more likely to occur during specific SWC clusters, the SWC clustering does not vary during the 24s QPP sequences, the goal of this work is to improve both the practical and theoretical understanding of different resting-state dynamics methods, thereby enabling investigators to better conceptualize and implement these tools for characterizing functional brain networks.

## Introduction

Resting-state functional MRI (rsfMRI) is a powerful tool for non-invasive examination of brain networks in healthy cognition, aging, disease, etc., but the traditional time-averaged approach only gives a summary of what happens over the course of the scan. Time-varying rsfMRI methods provide deeper insight into the brain’s functional architecture and the large-scale changes in activity that play out over the course of a scan (Keilholz et al., 2017; Preti et al., 2017) and have been shown to correlate with underlying neural activity (Tagliazucchi et al., 2012; Thompson et al., 2013; Keilholz, 2014) and behavior (Liegeois et al., 2019).

Several analysis methods have been proposed to capture time-varying activation and deactivation of functional networks throughout the brain. Some are based on the pattern of activity across the brain, while others specifically examine how the relationship between areas evolves over time. The temporal resolution of the technique is related to the length of the window used, which can range from a single TR to more than a minute. These analysis methods result in a time-varying series of matrices, which are often condensed into a handful of brain states using clustering techniques (Hutchison et al., 2013). Currently, there is little consensus about which analysis methods or parameterizations should be used, and as a result, researchers tend to choose methods somewhat arbitrarily. The wide variety of approaches hinders the comparison of findings obtained with different methods. Here we compare the outcomes for three different dynamic analysis methods coupled with k-means clustering and applied to the same data set, to provide insight into the similarities and differences of the results. A brief summary of each rsfMRI dynamic analysis method can be found in Table 1.

**TABLE 1:** Summary of dynamics methods.

Method	Sliding window correlation (SWC)	Phase synchrony (PS)	Co-activation patterns (CAP)	Quasi-periodic patterns (QPP)
Summary	Finds FC within brief (∼60s) windows. Repeating at each time-step along the full duration.	Finds the instantaneous phase of each voxel timeseries using a Hilbert transform. Calculates synchrony between phase angles of each voxel pair at each timepoint.	Cluster timepoints of BOLD data directly. Often performed on signal peaks above a given threshold (i.e., top 15%) for each voxel.	Searches the BOLD timeseries to find repeating spatiotemporal sequences of activation with the specified window length.
Parameter dependence	Window length	Robust	Robust	Window Length
Example references	Chang and Glover, 2010; Allen et al., 2014, 2018; Damaraju et al., 2014; Hindriks et al., 2016	Glerean et al., 2012; Yaesoubi et al., 2015; Cabral et al., 2017	Liu et al., 2013; Chen et al., 2015; Gutierrez-Barragan et al., 2019; Zhang et al., 2020	Majeed et al., 2011; Thompson et al., 2014; Yousefi et al., 2018; Abbas et al., 2019a; Yousefi and Keilholz, 2021

### Sliding Window

One of the most common techniques for examining time-varying rsfMRI is a sliding window (SWC) correlation analysis (Chang and Glover, 2010; Keilholz et al., 2013; Preti et al., 2017). Based on widely-used methods for calculating average functional connectivity (FC) as the correlation between brain areas over the course of the entire scan, in SWC, the fMRI timeseries is segmented using a time window of length *w* (typically 30–60s) and the correlation between pairs of brain regions is calculated for each window at every timestep.

### Phase Synchrony

Phase synchrony (PS) is similar to the sliding window approach, except the window length is reduced to a single timeframe by using Hilbert (Glerean et al., 2012; Cabral et al., 2017) or wavelet (Chang and Glover, 2010; Yaesoubi et al., 2015) transformations to obtain the instantaneous phase of the timeseries at each timepoint, then the PS between pairs of brain regions can be calculated. As with the sliding window approach, k-means clustering (Yaesoubi et al., 2015; Cabral et al., 2017) can then be applied to identify temporal dynamics.

### Co-activation Patterns

Another approach to the analysis of single timeframe rsfMRI is to examine co-activation patterns (CAP) (Liu et al., 2013; Chen et al., 2015). In contrast to the phase synchrony approach, CAP analysis identifies simultaneous occurrence of BOLD signal peaks or troughs in different brain regions independent of signal phase. The relationship between the BOLD signal and neural activity has been shown to arise from the temporally sparse events which form the basis of CAP analysis (Zhang et al., 2020). CAP analysis typically utilizes k-means clustering to identify different CAPs and this allows temporal dynamics to be identified and potentially compared to other time-varying rsfMRI methods including SWC and PS.

### Quasi-Periodic Patterns

Finally, one alternative to temporal clustering of single timeframes is to identify spatiotemporal patterns which occur repeatedly over sequences of frames (Majeed et al., 2011). Quasi-periodic patterns (QPPs) observed through this type of analysis represent a known dynamic feature of rsfMRI (Yousefi et al., 2018; Bolt et al., 2021) and have been shown to correlate with neural activity (Thompson et al., 2014). The strongest QPP typically consists of a sequence displaying a transition between a period of strong activation of default-mode network (DMN) and deactivation of sensory and attention networks to a period of DMN deactivation coupled with activation of sensory and attention networks (Abbas et al., 2019a; Yousefi and Keilholz, 2021).

We examine the relationship between brain states formed using SWC, PS, or CAP analysis, and investigate how each one classifies QPPs observed in the dataset. Our findings highlight commonalities and disparities across the methods and provide some insight into the sensitivity of various approaches.

## Materials and Methods

### Data and Preprocessing

The minimally preprocessed grayordinate and FIX de-noised rsfMRI of the HCP S900 release (Van Essen et al., 2012) consisting of 817 individuals with four complete rsfMRI scans (1,200-timepoints, TR = 0.72s) were downloaded and preprocessed for previous studies (Yousefi et al., 2018; Keilholz et al., 2020). In brief, each timeseries was demeaned and bandpass filtered (0.01–0.1 Hz), and global (white matter, cerebrospinal fluid (CSF), and gray matter) signals were regressed. The spatial dimension was reduced to 360 cortical parcels (Glasser et al., 2016) and each parcel’s timeseries was z-standardized. The first scan of the first day for each subject was used in all calculations of time-varying FC.

### Sliding Window Connectivity Analysis

Square 60s windows (83 timepoints), overlapping for all but a single timepoint, were utilized for the sliding-window analysis (Shakil et al., 2016). The Pearson correlation for each parcel-pair was calculated and Fisher transformed to normalize the variance during each 60s time-window from the first timepoint and at each ensuing time-step (1TR = 0.72s) with windows overlapping except for a single timepoint. A symmetrical 360 × 360 matrix of pairwise values for all brain regions was obtained for each window (1,117 per scan = 1,200 total timepoints–window length) and a vector-by-time matrix containing a single value for each of the 64,620 parcel-pairs (equivalent to the lower triangle of the 360 × 360 matrix) at each timepoint served as the input for the clustering algorithm to define brain states.

### Phase Synchrony Analysis

The instantaneous phase was calculated at every timepoint by taking the Hilbert transform for each parcel. The phase synchrony between each parcel-pair was then computed as the cosine of the difference in phase angles at each timepoint. A vector-by-time matrix of 64,620 pairwise phase synchrony values was obtained for each timepoint and used as the input for the clustering algorithm.

### Co-activation Pattern Analysis

The original CAP analysis applied an activation threshold (often top 15% or z-score > 1) to a seed region and averaged BOLD frames across all suprathreshold timepoints (Liu and Duyn, 2013). Further work extended CAP analysis to a more data driven, whole-brain approach by performing k-means clustering directly on the BOLD timeseries data (Liu et al., 2013). For the present study we performed k-means clustering of the 360 parcels at each timepoint. In order to display cluster centers in a comparable format to the other methods, after clustering, the 360 elements of each centroid were multiplied with themselves generating a 360 × 360 outer-product matrix for each cluster center.

### K-Means Clustering

Clustering was performed in MATLAB using the k-means function with k = 5 as the cluster number, correlation distance as the distance metric, and 30 replications per run for each of the SWC, PS, and CAP methods. Each of these methods generated a 2D vector-by-time matrix of values for all 360 parcels (CAP) or 64,620 parcel-pairs (SWC/PS) at every timepoint (1,200/scan for PS/CAP) or every time window (1,117/scan for SWC). For each dynamic analysis method, the vector by time matrices were initially clustered for each individual scan. The cluster centroids (means) from all individual scans were then clustered by k-means using the same parameters to generate group-level cluster centroids. The group-level centroids were the used to initialize clustering of all timepoints across all individuals. This iterative clustering process has been utilized in previous studies of brain dynamics (Allen et al., 2014, 2018) and greatly reduces the computational time and resources required for large datasets.

Selecting an appropriate number of clusters is a critical part of this type of dynamic analysis. Larger numbers of clusters can potentially provide more information but increasing the cluster number greatly increases computation time and can make interpretation more challenging by increasing complexity and reducing the distinction between clusters. For this study, preliminary results were generated using a range of 3–10 clusters on a subset of 20 subject scans (but otherwise the same methods described above). These preliminary results are displayed in Supplementary Materials for k = 3, 7, and 10 clusters. Based on these initial observations, k = 5 clusters were selected as a sufficient number to provide an effective comparison between the different dynamic analysis methods. While the ideal cluster number may vary for the different approaches, it was important to use a consistent number for this comparative analysis.

### Comparing the Methods

The resulting clusters, termed brain states, for each dynamic analysis method were visualized by plotting of the cluster centroids as 360 × 360 pairwise matrices with the parcels grouped into seven cortical functional networks as defined by Yeo et al. (2014). Weighted cluster averages were computed for each method as a comparison to time-averaged functional connectivity by taking the mean of the five cluster centroids weighted by the state occurrence rates. The clusters from each method were also compared against each other using several dynamic metrics, including the occurrence rate of each state, the probability of a transition from one state to each of the other states [after concatenating across subjects and ignoring within-state transitions; method described by Cornblath et al. (2020)], and the mean dwell time spent within one state before a transition to another state. The state composition was also compared for each method to determine whether timepoints identified as belonging to a specific brain state by one method were likely to belong to a specific state identified by one of the other methods.

### Quasi-Periodic Patterns

Quasi-periodic patterns analysis identifies repeating spatiotemporal sequences of a specified duration using a pattern finding algorithm (Majeed et al., 2011). The QPP can be thought of as a sequence of specific brain states that repeats over time. For this analysis, a duration of 33 timepoints (24s) was used based on previous work showing prominent QPPs with a duration of 20-24s occurring in resting-state data (Abbas et al., 2019a). Over the full dataset a total of 6,717 occurrences (defined as 33-timepoint sequences with a correlation of *r* ≥ 0.3 to the QPP template) of the strongest QPP were identified. The QPP was used to compare across dynamic analysis methods by determining the probability of each of the 33 timepoints in the QPP sequence being identified as belonging to a specific brain state using each method.

### Statistical Testing

Several comparisons between analysis methods were tested for statistical significance using MATLAB functions. Permutation tests were performed to quantify the significance of sequential state transitions differing from chance in a method similar to the one described by Cornblath et al. (2020). A total of 100,000 permutations were used to calculate the probability distribution of transitioning to the next (different) state from the current state given the overall occurrence rates of each state with Bonferroni correction for multiple comparisons. The Kruskal-Wallis test was performed to quantify differences in the distribution of subject-mean dwell times across states within and between each of the three analysis methods. The Mann-Whitney test was performed for further pairwise comparisons of dwell times using Bonferroni correction for multiple comparisons. Chi-square goodness of fit tests were used to test for overlap in the composition of states identified by the different analysis methods. The occurrence rates of each state over all 980,400 timepoints were input as the expected frequency and compared against observed frequencies to determine overlap in state composition. The same Chi-square procedure was used to quantify the likelihood that timepoints belonging to a QPP would be clustered into a specific state.

## Results

**FIGURE 1:**
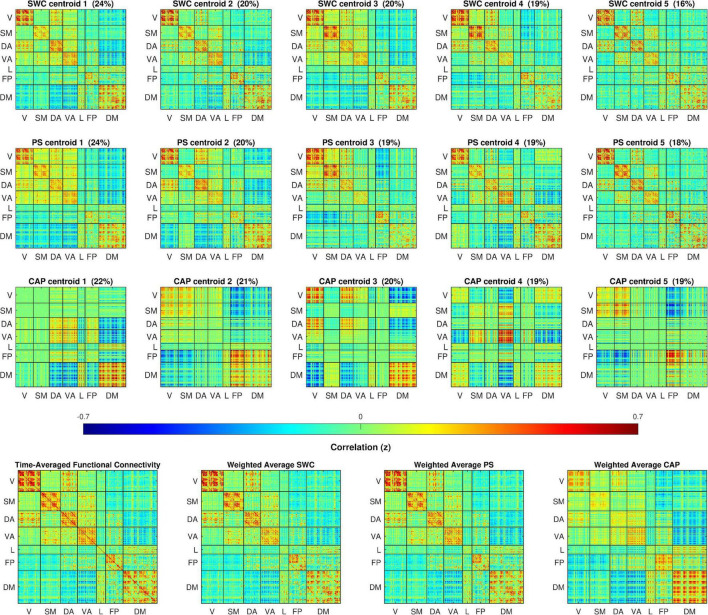
The top three rows plot cluster centers colored by z-scores of correlation, cosine, and BOLD (BOLD amplitude squared) between parcel-pairs of sliding window connectivity (SWC), phase synchrony (PS), and co-activation pattern (CAP) clusters respectively. The clusters are sorted by occurrence rate (% shown above each centroid). The 360 spatial parcels are sorted into seven major functional networks identified by Yeo et al. (2014). The bottom row displays the time-averaged FC for the full dataset and the mean across cluster centers weighted by the occurrence rates for each dynamic method. The cluster weighted averages are correlated with time-averaged FC with *r* = 0.994, 0.998, and 0.873 for SWC, PS, and CAP methods respectively.

The cluster centers resulting from k-means clustering for each of the dynamic analysis methods are illustrated in Figure 1. To enable direct visual comparison, every plot in Figure 1 appears on the same color scale and the cluster centers are z-score normalized and scaled by the standard deviation of the time-averaged FC. The cluster centers for each method appear qualitatively similar to previous findings (Allen et al., 2014; Damaraju et al., 2014; Yaesoubi et al., 2015). Clear differences are evident between the three methods, particularly between CAP and the other two. The SWC and PS centroids all somewhat resemble the plot of time-averaged FC over the entire dataset, except with some variations in the levels of correlation within major networks and considerable variation in correlations between major networks. Qualitatively, the PS centroids appear quite similar to SWC centroids but with slightly greater variation between the states. In stark contrast are the CAP centroids, which appear to capture periods dominated by activation of specific networks and display a much greater degree of variation across states. The weighted cluster averages for each method closely resemble, and were strongly correlated to, the time-averaged FC with *r* = 0.994, 0.998, and 0.863 for SWC, PS, and CAP averages respectively.

**FIGURE 2:**
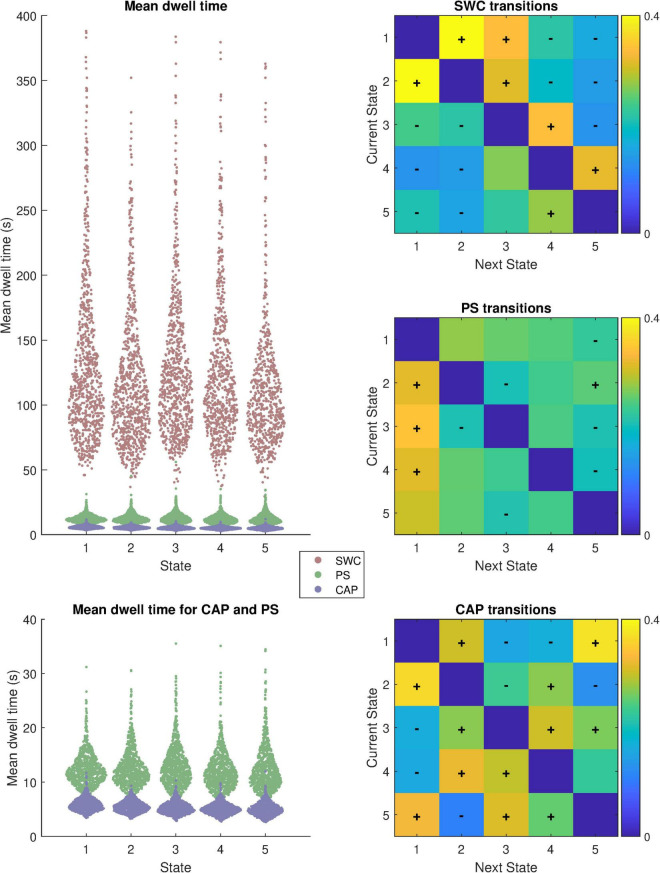
Summary of dynamics metrics; Distribution of mean dwell time between state transitions for each state in each subject (*N* = 817) and transition probability matrices from each state to the next. Mean dwell time is displayed with and without SWC included. The “+” and “−” symbols overlaid on the transition probability matrices indicate transitions that are more or less likely, respectively, than chance to occur based on permutation tests (*p* < 0.05; Bonferroni corrected for multiple comparisons).

Further differences between the methods are highlighted by the metrics displayed in Figure 2 which highlights the distributions of mean dwell time for each subject within each state before a transition and also plots matrices of transition probability from one state to another. The distributions of mean dwell times differed substantially for the different analysis methods (Kruskal-Wallis Chi-squared = 10,722; *p* < 0.05) with SWC brain states exhibiting much longer dwell times than PS (Mann-Whitney z = 35.2; *p* < 0.05) or CAP (*z* = 35.2; *p* < 0.05). For SWC, state 2 had the shortest mean dwell time of 139 s (median 114.5 s) while state 1 had the longest 163 s (median 128.5 s). Mean dwell times for PS states ranged from 12 to 13 s and were significantly longer (*z* = 34.9; *p* < 0.05) than CAP states which ranged from 5 to 6 s.

The transition probability matrices show that each analysis method produces non-random state sequences. The plus and minus signs overlayed on the heatmap indicate transitions to the next (different) state from the current state that occur more (“+”) or less (“−“) frequently than if state transitions occurred randomly (*p* < 0.05; Bonferroni corrected for 20 comparisons per analysis method).

**FIGURE 3:**
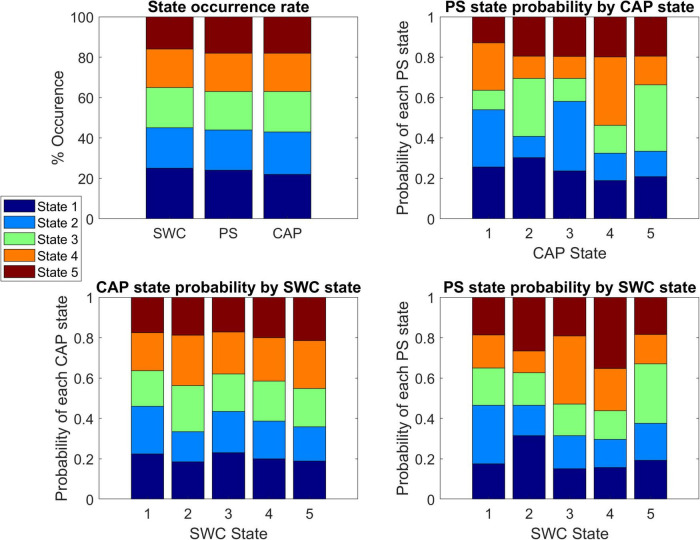
Comparison of state composition across dynamics methods. Top-left shows the state occurrence rate of each analysis method with states sorted from highest occurrence (state 1) to lowest occurrence (state 5). Top-right shows the probability of a timepoint that belongs to a specified co-activation pattern (CAP) state corresponding to each of the five phase synchrony (PS) states. The bottom panels show the probability of a timepoint that belongs to a specified CAP state (Bottom-left) or PS state (Bottom-right) corresponding to each of the five sliding window connectivity (SWC) states.

Figure 3 displays the state occurrence rates for each method and the probability that a timepoint belonging to a state from one method corresponds to a specific state identified by another method. For comparison to SWC, the observed CAP or PS states corresponding to every timepoint within the SWC windows were included for every SWC window of each state. Each CAP state showed a significant deviation from the expected (null) distribution in the frequency of belonging to specific PS states (Chi-squared values = 53, 72, 85, 82, 74 respectively; *p* < 0.05 for each, Bonferroni corrected). Similarly, each SWC window showed a significant deviation from the expected (null) distribution in the frequency of observed PS states (Chi-squared values = 31, 58, 81, 113, 40 respectively; *p* < 0.05 for each, Bonferroni corrected). However, only SWC windows in state 2 showed a significantly non-random distribution of observed CAP states (Chi-squared = 23; *p* < 0.05, Bonferroni corrected).

**FIGURE 4:**
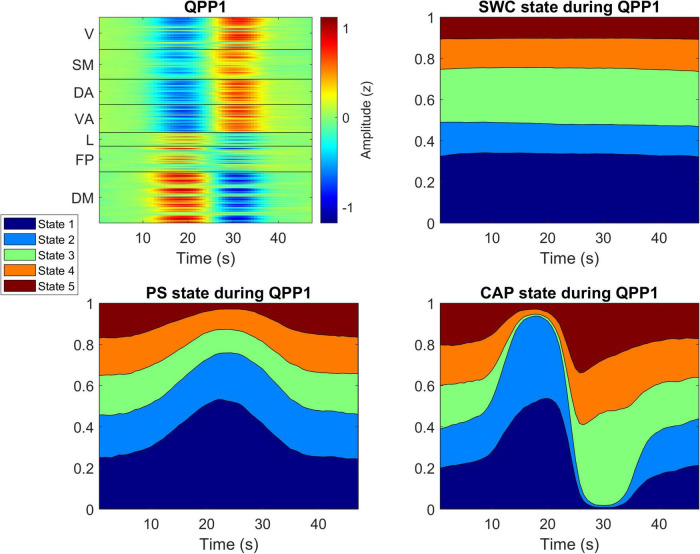
Comparison of most likely states during QPP1. Top-left shows spatiotemporal QPP1 template with timepoints on the x-axis and space (Glasser’s 360 parcels) on the y-axis and colors corresponding to z-score BOLD signal amplitude. The other three panels show the same timepoints on the x-axis with the likelihood of being in each state plotted on the y-axis with the colors corresponding to the five brain states identified by each dynamic analysis method. A video representation of the QPP1 template mapped to a 3D brain surface can be found in Supplementary Materials.

The template of the strongest QPP (termed QPP1), consisting of the average of all identified recurrences of the 33-timpoint spatiotemporal pattern padded by the 17 timepoints before and after, is shown in Figure 4. In agreement with previous findings (Yousefi et al., 2018; Abbas et al., 2019a), QPP1 consists of a period of high DMN activation followed by a transition to a period of high sensory (visual and somatomotor) and attention (both dorsal and ventral) network activation with strong anti-correlation of the DMN to sensory and attention networks evident during each phase. A video representation of the QPP1 template mapped to a 3D brain surface can be found in Supplementary Materials. A mean (± standard deviation) of 8.2 ± 3.1 QPP1 sequences were observed per subject with a maximum of 18 QPP1 sequences in one subject and three subjects with no QPP1 sequences detected.

Figure 4 also displays plots of the probability of those same timepoints belonging to the 5 brain states identified for each of the three dynamic analysis methods. These plots highlight the relationship between the methods. For the CAP analysis, states 1 and 2 significantly increase in likelihood (Chi-squared = 105; *p* < 0.05) during high DMN activation, and states 3, 4, and 5 each increase significantly (Chi-squared = 74; *p* < 0.05) in likelihood during high activation of sensory and attention networks. For PS, the likelihood of state 1 increases while that of state 2 decreases for the entirety of the QPP, peaking in the middle (Chi-squared = 51; *p* < 0.05). Finally, unlike with the other two methods, SWC state probabilities remain nearly constant throughout the QPP with no significant deviations at any timepoint, although the QPP sequences did show increased likelihood of occurring during SWC states 1 and 3 (Chi-squared = 11; *p* < 0.05).

The results shown in Figures 1–4 were obtained using global signal regression (Power et al., 2015). These analyses were replicated on the same data with gray-matter signal regression removed from the pre-processing with the results displayed in Supplementary Materials (Supplementary Figures 1–4). While the cluster centers in Supplementary Figure 1 differ visually from those in Figure 1, the dwell times (Supplementary Figure 2), overlap in cluster occurrence (Supplementary Figure 3), and state trajectories during QPPs (Supplementary Figure 4) are all qualitatively similar without gray-matter signal regression with no apparent differences in the relationships between the analysis methods.

The Supplementary Materials also contain results for each analysis method performed on a subset of the data (*N* = 20) using k = 3, 7, 10 clusters. Supplementary Figures 5–7 display cluster centers (comparable to Figure 1) for each method with these alternate cluster numbers. CAP centers show consistently high within-network activity and become increasingly specific to single networks with increasing cluster number, whereas SWC and PS centers capture different cross-network configurations which display increasingly subtle variations as the cluster number is increased. Supplementary Figures 8–10 show the categorization of QPP timepoints (comparable to Figure 4) for each method with the different cluster numbers. While the plots appear less smooth due to the smaller dataset, the relationship between the QPPs and clustering appears consistent across cluster number for each method. Supplementary Figure 11 displays the average sum of within-cluster distances for *k* = 2:10 for each method using the same subset of the data. The plot displays smooth curves for each analysis method and does not indicate a clear optimal selection for the cluster number.

## Discussion

One of the challenges involved in interpreting time-varying connectivity metrics in the current literature is that each approach is sensitive to a different aspect of the brain’s dynamics. Here for the first time, we look for an explicit relationship between these different dynamic analysis methods applied to the same dataset, providing a basis for understanding the relationship between different analysis approaches.

The results provide a detailed comparison between brain states obtained with the four dynamic analysis methods: SWC, PS, CAPs, and QPPs. Perhaps unsurprisingly, the differences between these methods are shown to result in prominent differences in brain states identified by k-means clustering despite each method closely reproducing time-averaged FC when the states are averaged sequentially (as shown in Figure 1). In particular, the results indicate that SWC with a 60s window length may not provide the necessary temporal resolution to capture important features of brain dynamics, such as QPPs, that occur on shorter time scales. This is clearly illustrated by the much longer dwell times for SWC states, which might be expected from the temporal smoothness caused by the 60s windows overlapping by all but a single timepoint of offset. While the 60s window length is a common choice for SWC, the use of a shorter window would likely produce a more similar result to the PS method, however there is evidence that the use of very short SWC windows can potentially introduce spurious correlations (Leonardi and Van De Ville, 2015). See Shakil et al. (2016) for an extensive evaluation of SWC parameters for dynamic analysis.

Meanwhile PS and CAP analysis both provide superior temporal resolution at the level of a single timepoint, but each produces quite different brain states following k-means clustering due to the underlying features each method is sensitive to. This point is demonstrated by how each of these two methods captures QPPs. PS is sensitive to correlations between regions and frequently categorizes the entire QPP into brain state 1 because of the prominent anti-correlation of the DMN to sensory and attention networks that characterizes the QPP. Intriguingly the probability of PS state 1 is highest during the transition period between the QPP peak and trough, likely indicating that this transition is the period of greatest coherence of the regional signal trajectories. Meanwhile, because CAP analysis is sensitive to signal amplitude the first phase of the QPP, when DMN is activated, is categorized into states 1 and 2 while the second phase of the QPP, when sensory and attention networks are activated, is categorized into states 3, 4, and 5 but almost never states 1 or 2. This finding could potentially explain the unexpected result of longer dwell times for PS compared to CAP as each QPP results in a greater number of CAP state transitions than PS state transitions. A similar relationship is observed when the data is analyzed without global signal regression. The QPPs are frequently categorized into a single PS state (especially during the mid-QPP phase transition period) while each phase of the QPP is mostly categorized into different CAP states and SWC states do not make any specific transitions during the QPPs.

### How to Choose the Best Dynamic Analysis Method for a Study

Sliding window connectivity may still be advantageous relative to time-averaged functional connectivity for comparisons between groups (Damaraju et al., 2014; Hindriks et al., 2016; Menon and Krishnamurthy, 2019) and window lengths shorter than the 60s used for this study may produce results closer to the PS method. However, PS and CAP methods do provide noteworthy advantages for dynamic analysis. In additional to capturing information at shorter time scales, each can potentially be performed without an arbitrary window length selection. While the CAP analysis for this study did not employ an activation threshold, performing clustering directly on the BOLD data, seed-based CAP analysis commonly utilizes an arbitrarily selected threshold that could influence the results (Liu et al., 2013; Chen et al., 2015; Gutierrez-Barragan et al., 2019; Zhang et al., 2020). Likewise, PS does not require the investigator to set any specific parameters, but the data is commonly bandpass filtered (Cabral et al., 2017) into frequency bands (typically 0.01–0.1 hz or lower) which is likely to reduce sensitivity to dynamics at higher frequencies. CAP analysis also offers the advantage of reduced dimensionality and computation time given the clustering is performed on data from N parcels rather than (N-1)*N/2 parcel pairs. However, Cabral et al. (2017) have shown that PS (and potentially SWC) analysis can be effectively performed on the leading eigenvector of the parcel pairs, reducing the dimensionality to the same number N and producing similar results.

Investigators may also wish to consider performing QPP analysis for examining brain dynamics as this method has demonstrated clear neural correlates (Thompson et al., 2014), reproducibility across individuals (Yousefi et al., 2018) and across species (Belloy et al., 2018a), and the potential to differentiate patient groups from controls (Abbas et al., 2019b). Further, as PS and CAP analysis have now been shown to be sensitive to QPPs, it is possible that regression of one or more QPPs could benefit a PS or CAP analysis by uncovering additional features that might be obscured by the widespread spatial changes of the QPP (Belloy et al., 2018b; Yousefi and Keilholz, 2021). Meanwhile, for studies explicitly seeking to identify dynamic changes occurring on slower time scales, the SWC method could be advantageous specifically because it is less sensitive to QPPs that could potentially confound the hypothesized differences. Indeed, there is evidence suggesting that SWC methods featuring window lengths ≥30s perform better than shorter windows or single timepoint methods at segmenting different cognitive tasks (Xie et al., 2019).

Finally, there is recent evidence that the correlation between BOLD signal and underlying neural activity arises specifically from the type of peak amplitude events utilized for CAP analysis (Zhang et al., 2020), potentially making CAPs the most sensitive method for studying neural brain dynamics. However, more research is certainly needed to elucidate the relationship between BOLD signal dynamics and neural activity.

### Further Considerations and Limitations

One significant limitation of this study is that only k = 5 clusters/brain states were fully tested. Choosing an appropriate number of states for a particular study is important and can pose a challenge as there is not a well-established number of brain dynamics features and no perfect method for determining the optimal number to select. Some studies (Allen et al., 2014, 2018; Damaraju et al., 2014; Gutierrez-Barragan et al., 2019) have employed the “elbow method” in which the ratios of within-cluster distances to between-cluster distances are plotted for a series of k cluster numbers (see Supplementary Figure 11) and a number k is selected which protrudes from the plot such that a greater contribution to optimizing the clustering is apparent for k than for k + 1. However, the number of clusters featuring optimized distances may not always be the optimal number for investigating brain dynamics and ideally studies will investigate a range of k clusters as shown here in the Supplementary Materials for a subset of the data (*N* = 20). For this study, the subset results do not indicate an optimal cluster number for any method using the elbow criterion but do appear consistent across a range of 3–10 clusters with each analysis method displaying common features and clustering QPP sequences in a consistent manner.

An important methodological consideration for this type of analysis is the distance metric employed for k-means clustering. While the correlation distance was used for the present study, some have suggested that L1 distance may offer advantages for this type of analysis (Allen et al., 2014). However, in performing this study the L1 distance was found to require greater computation time than the L2 or correlation distance metrics, and in limited testing the correlation distance appeared to offer the most qualitatively consistent results.

## Data Availability Statement

The raw data supporting the conclusions of this article will be made available by the authors, without undue reservation, to any qualified researcher.

## Ethics Statement

Ethical review and approval was not required for the study on human participants in accordance with the local legislation and institutional requirements. Written informed consent for participation was not required for this study in accordance with the national legislation and the institutional requirements.

## Author Contributions

EM designed and executed the study and created the figures. BY performed the preprocessing. BY, XZ, AK, and SK discussed the ideas and gave critical insights and feedback on methods. All authors contributed to the article and approved the submitted version.

## Conflict of Interest

The authors declare that the research was conducted in the absence of any commercial or financial relationships that could be construed as a potential conflict of interest.

## Publisher’s Note

All claims expressed in this article are solely those of the authors and do not necessarily represent those of their affiliated organizations, or those of the publisher, the editors and the reviewers. Any product that may be evaluated in this article, or claim that may be made by its manufacturer, is not guaranteed or endorsed by the publisher.
