# Supplementary material for: Comparison of Resting-State Functional MRI Methods for Characterizing Brain Dynamics
Source: Front Neural Circuits. 2022 Apr 4;16:681544. doi: 10.3389/fncir.2022.681544 (PMC9013751; doi:10.3389/fncir.2022.681544)
Supplement: Supplementary file 2 [file Data_Sheet_1.DOCX]

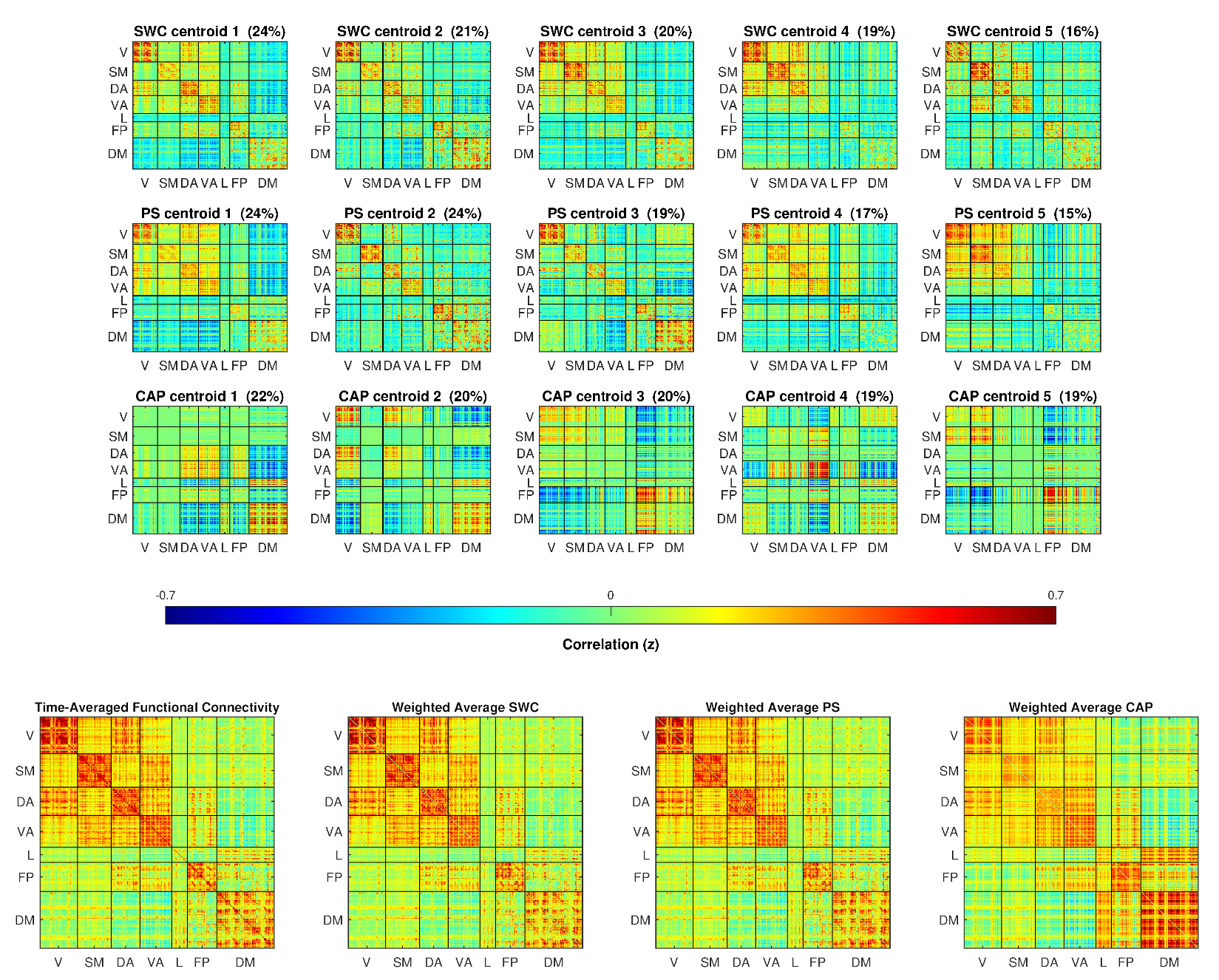


**Figure S1** Cluster centers without global signal regression; colored by correlation (z) of parcel-pairs compared across dynamic methods. The clusters are sorted by occurrence rate (% shown above each centroid). The 360 spatial parcels are sorted into 7 major functional networks identified by Yeo et al. (Yeo, Krienen et al. 2014). The bottom row displays the time-averaged FC for the full dataset and the mean across cluster centers weighted by the occurrence rates for each dynamic method. The cluster weighted averages are correlated with time-averaged FC with r = 0.993, 0.998, and 0.804 for SWC, PS, and CAP methods respectively.


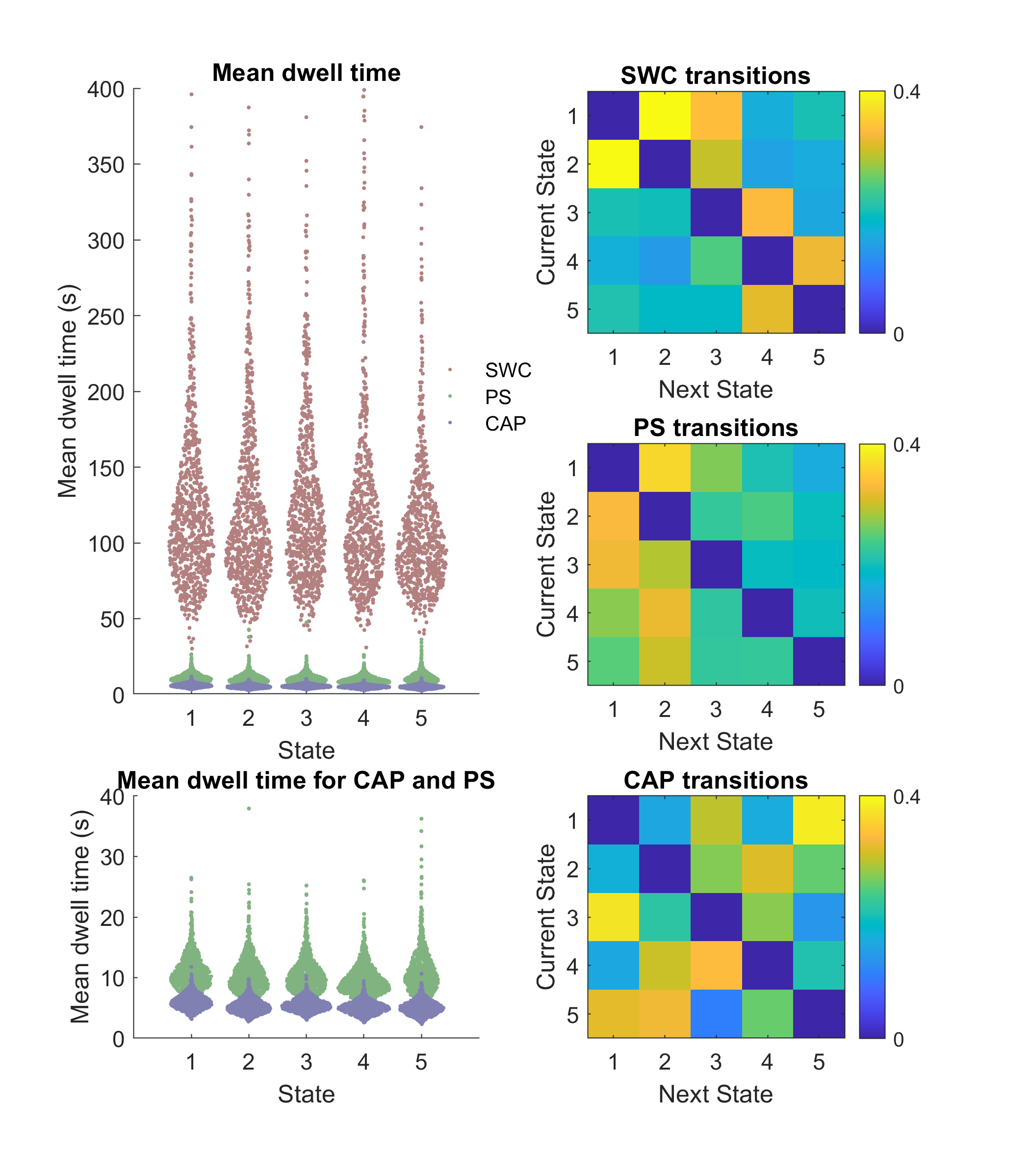


**Figure S2** Summary of dynamics metrics without global signal regression; Distribution of mean dwell time between state transitions for each state in each subject (N=817) and transition probability matrices from each state to the next. Mean dwell time is displayed with and without SWC included.


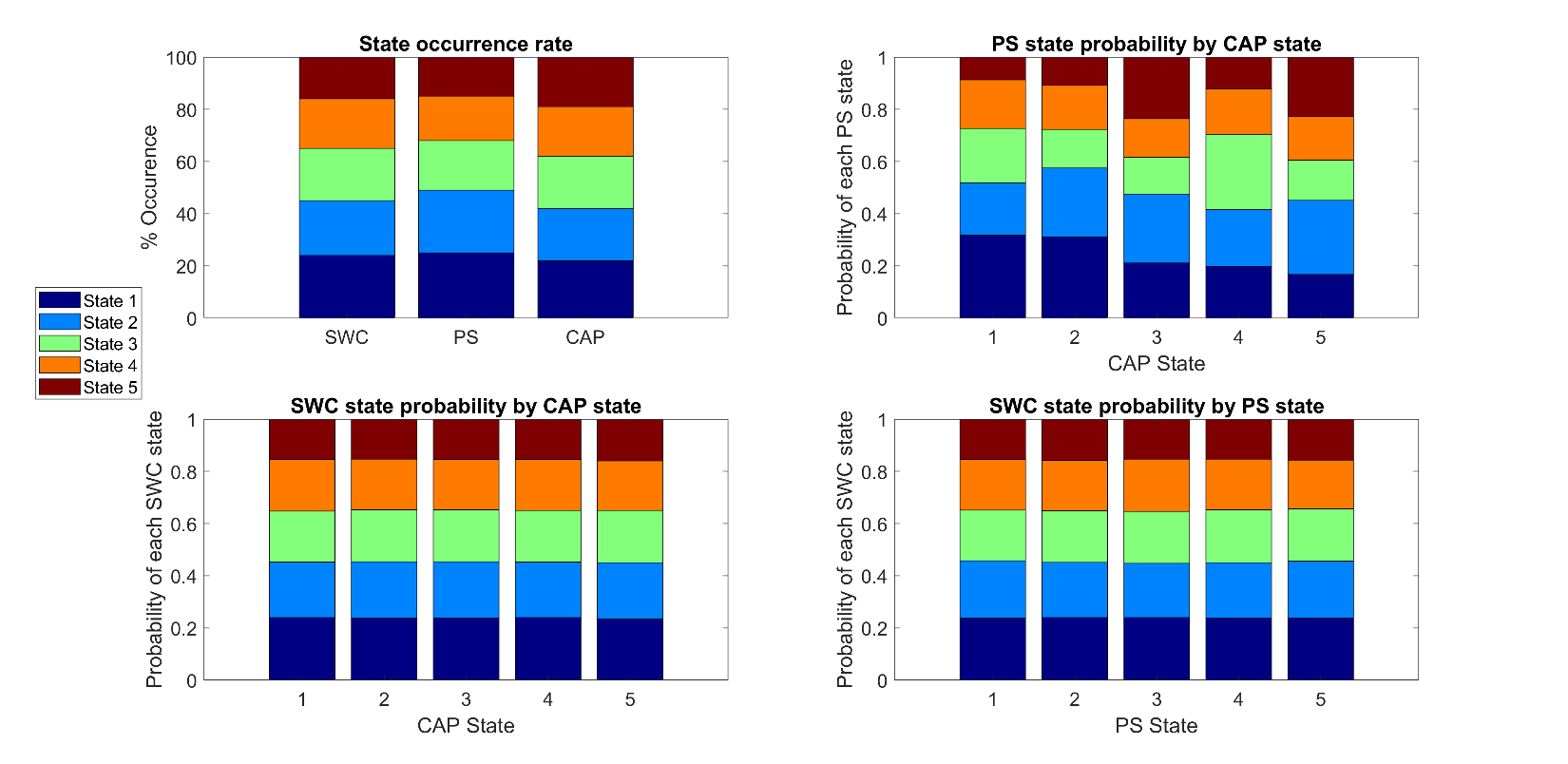


**Figure S3** Comparison of state composition across dynamics methods without global signal regression. Top-left shows the state occurrence rate of each analysis method with states sorted from highest occurrence (state 1) to lowest occurrence (state 5). Top-right shows the probability of a timepoint that belongs to a specified CAP state corresponding to each of the 5 PS states. The bottom panels show the probability of a timepoint that belongs to a specified CAP state (Bottom-left) or PS state (Bottom-right) corresponding to each of the 5 SWC states.


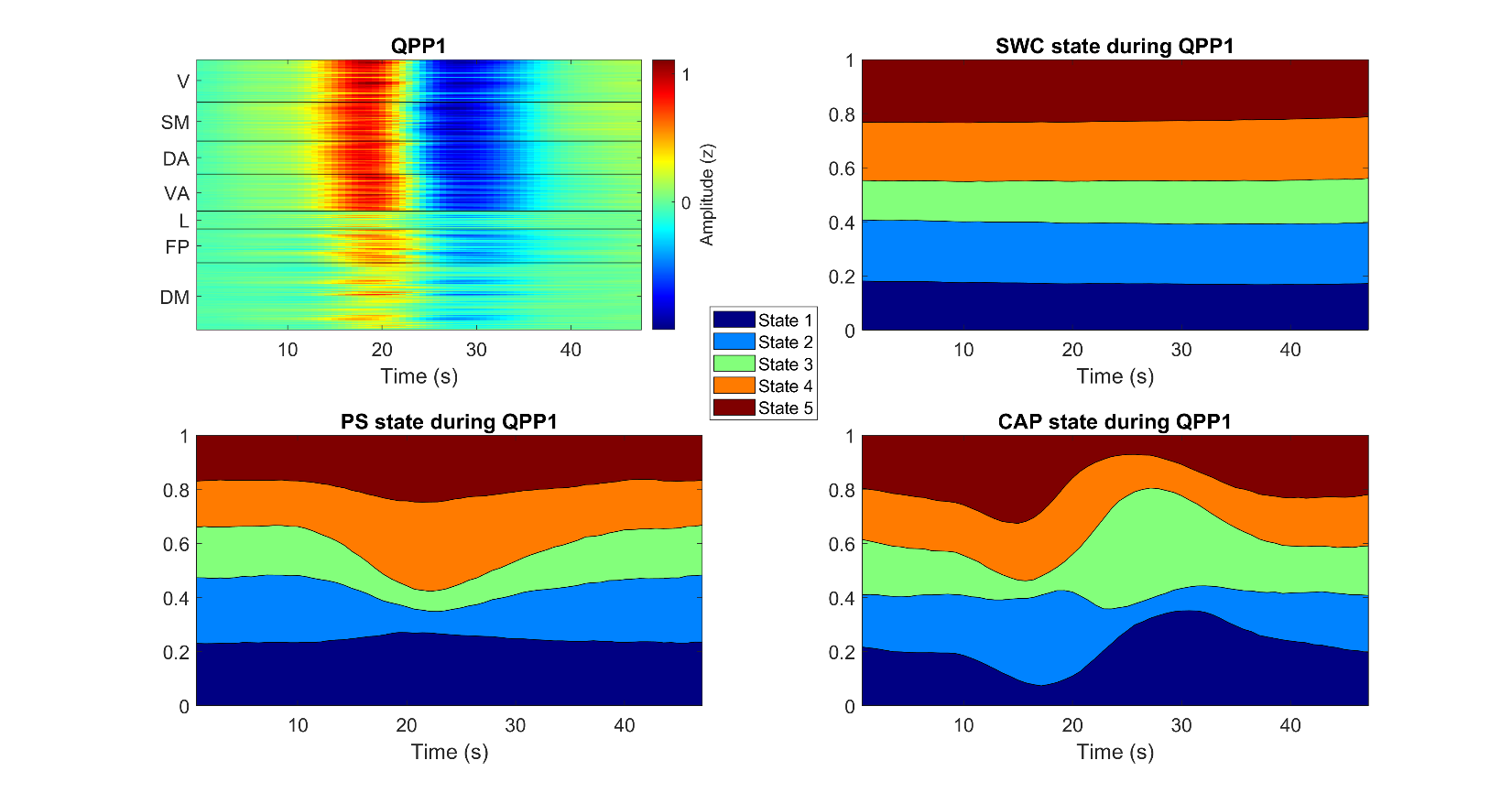


**Figure S4** Comparison of most likely states during QPP1 without global signal regression. Top-left shows spatiotemporal QPP1 template with timepoints on the x-axis and space (Glasser’s 360 parcels) on the y-axis and colors corresponding to z-score BOLD signal amplitude. The other 3 panels show the same timepoints on the x-axis with the likelihood of being in each state plotted on the y-axis with the colors corresponding to the 5 brain states identified by each dynamic analysis method.


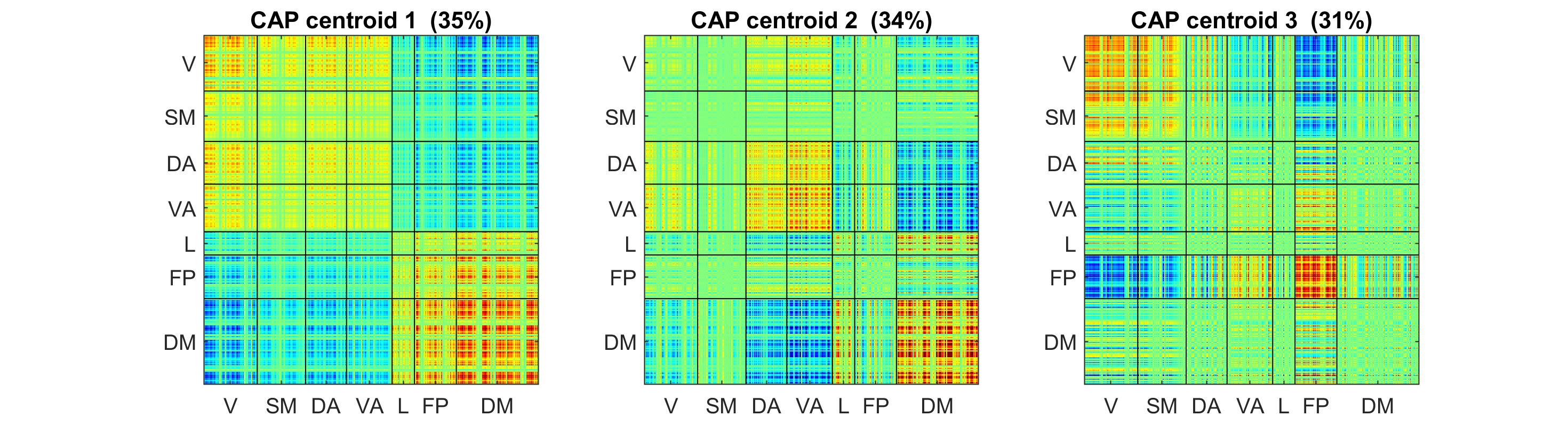

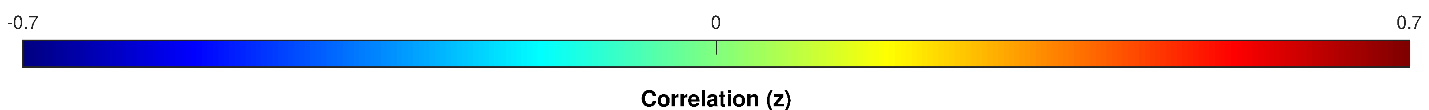

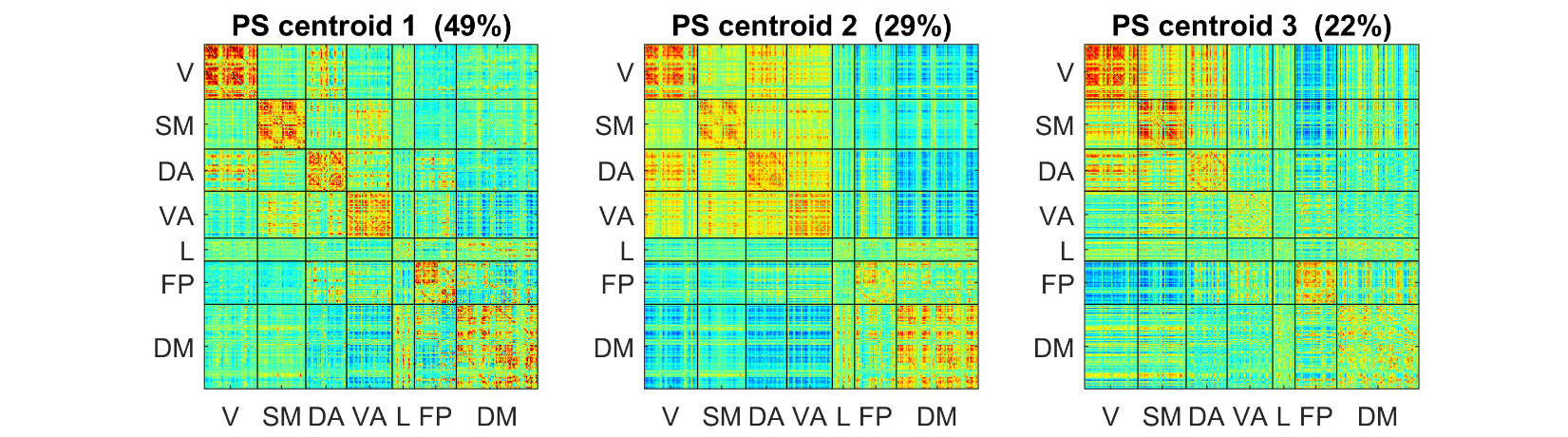

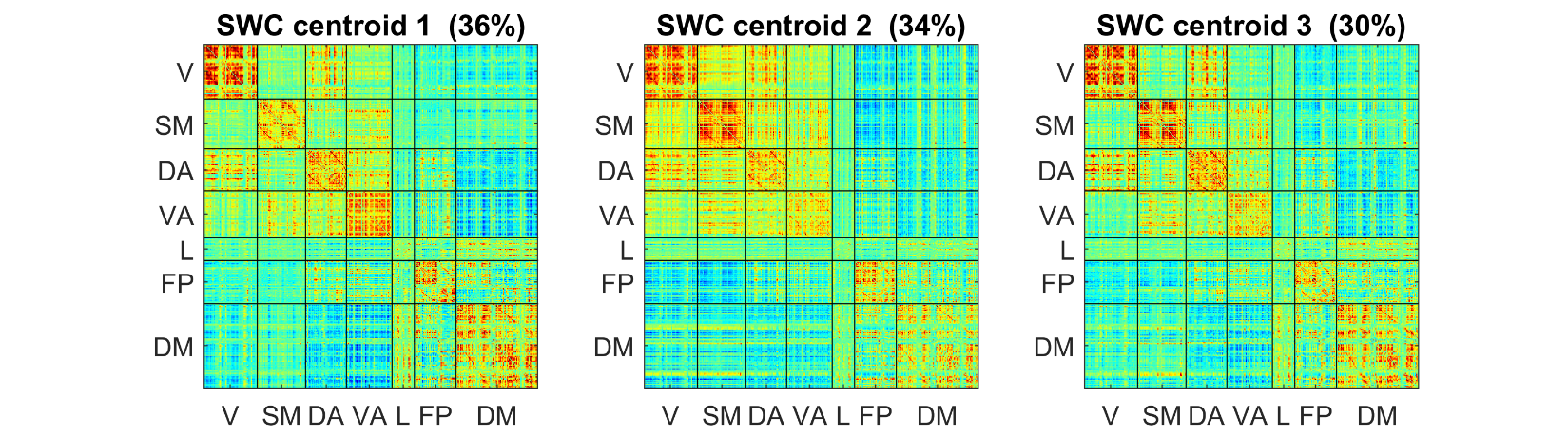


**Figure S5** Cluster centers with k=3 clusters for a subset of the data (N=20); colored by correlation (z) of parcel-pairs compared across dynamic methods. The clusters are sorted by occurrence rate (% shown above each centroid). The 360 spatial parcels are sorted into 7 major functional networks identified by Yeo et al. (Yeo, Krienen et al. 2014).


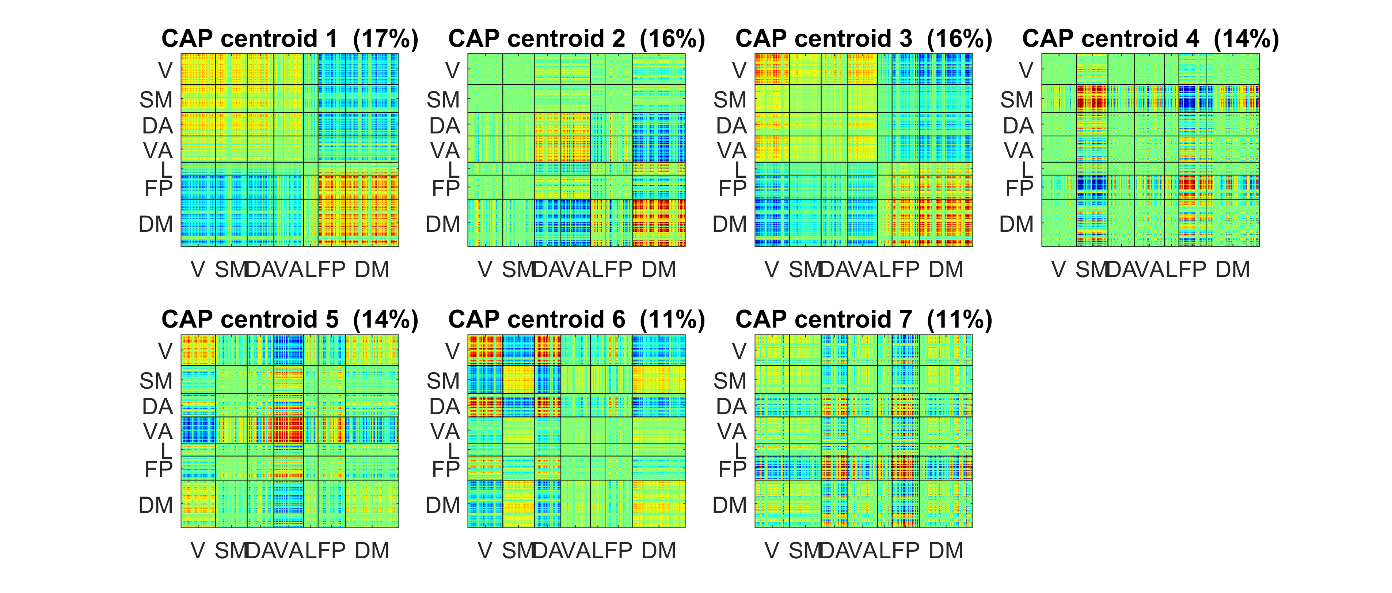

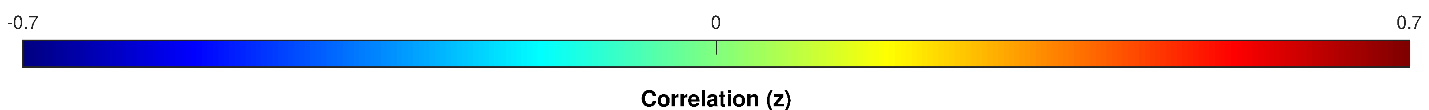

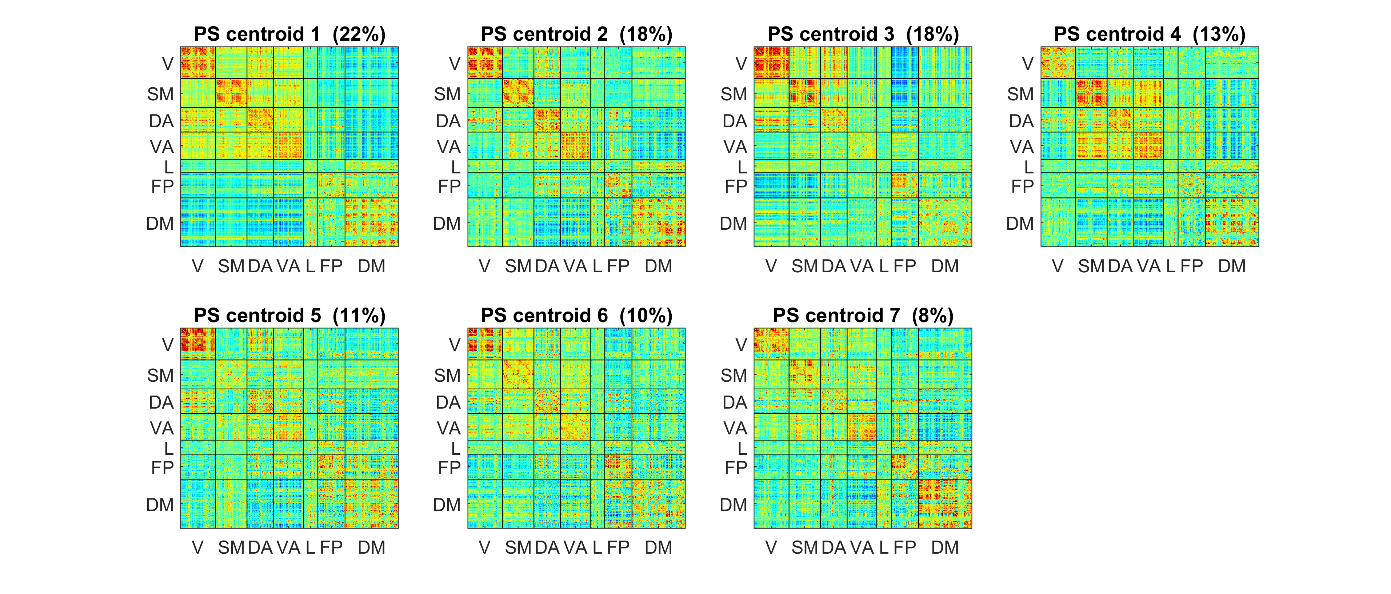

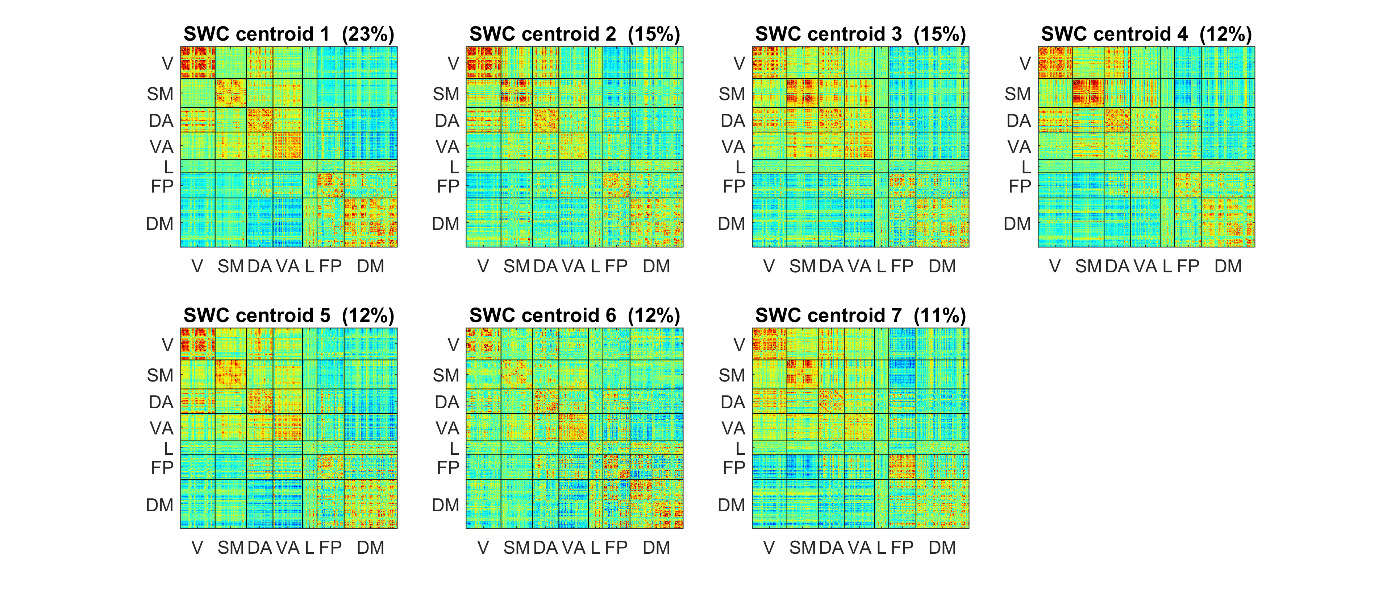


**Figure S6** Cluster centers with k=7 clusters for a subset of the data (N=20); colored by correlation (z) of parcel-pairs compared across dynamic methods. The clusters are sorted by occurrence rate (% shown above each centroid). The 360 spatial parcels are sorted into 7 major functional networks identified by Yeo et al. (Yeo, Krienen et al. 2014).


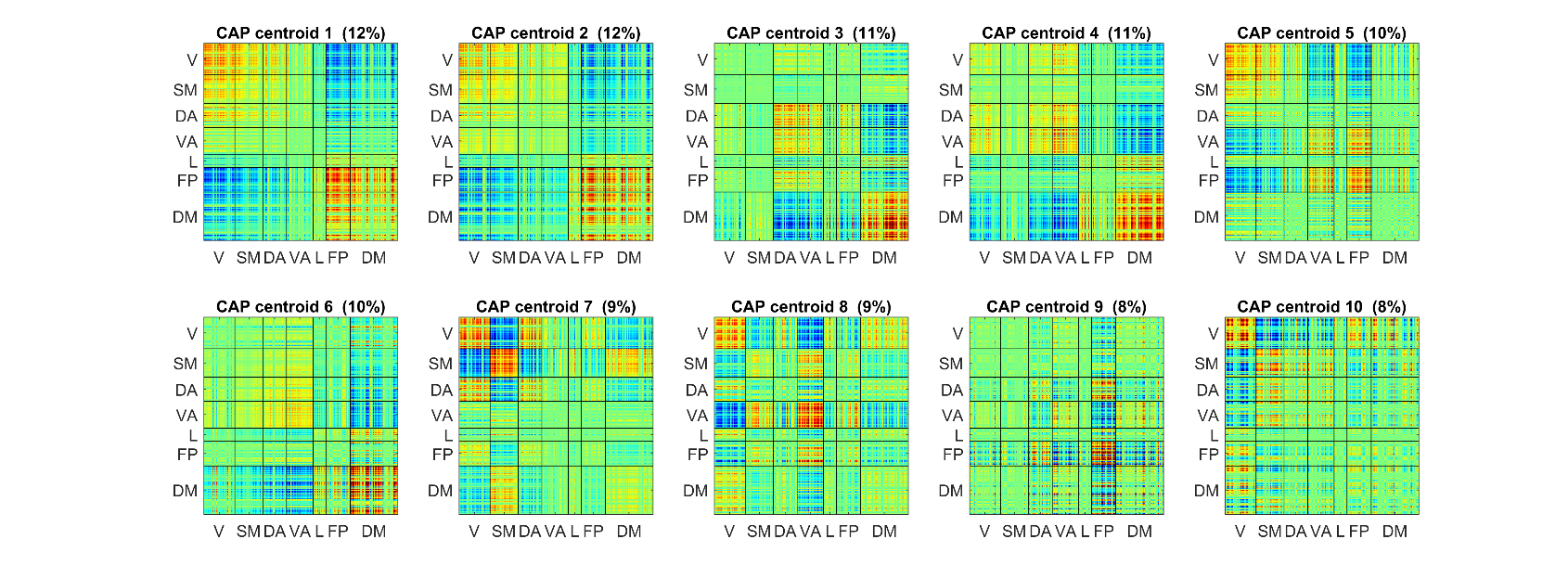

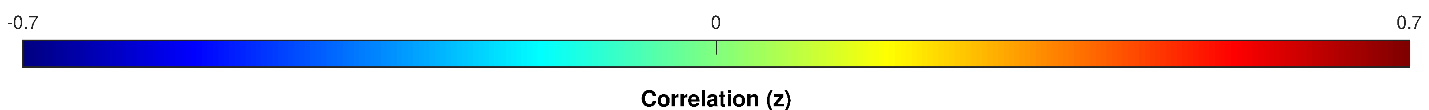

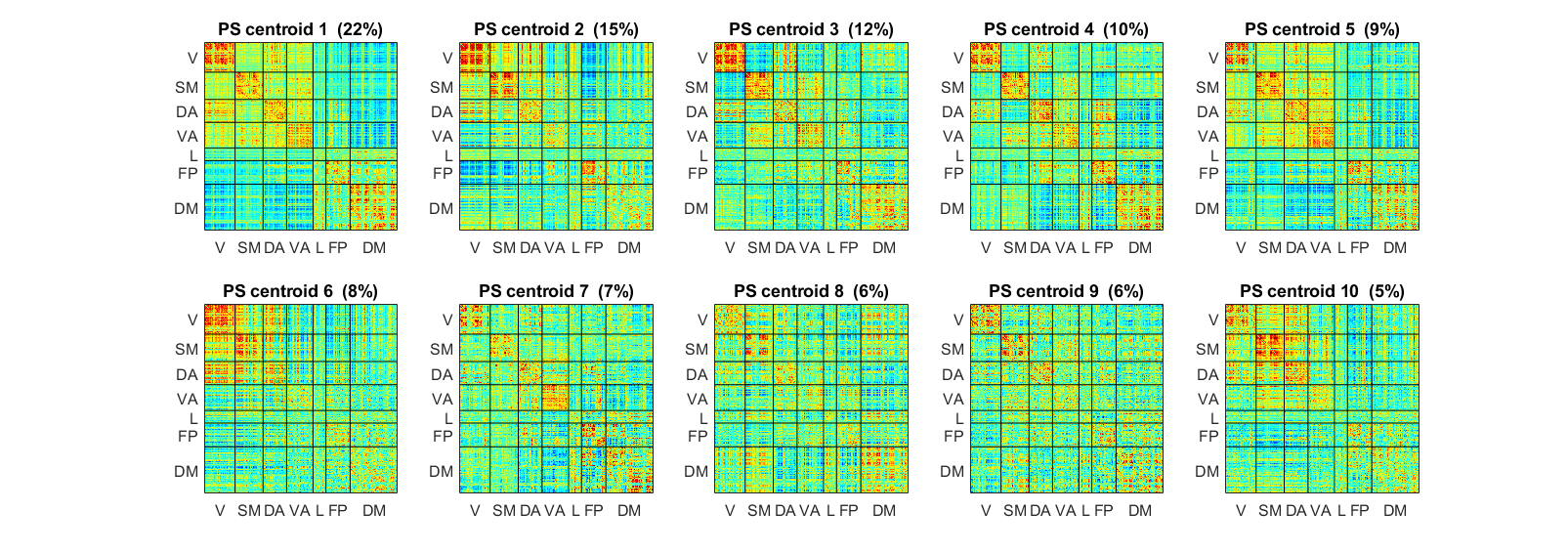

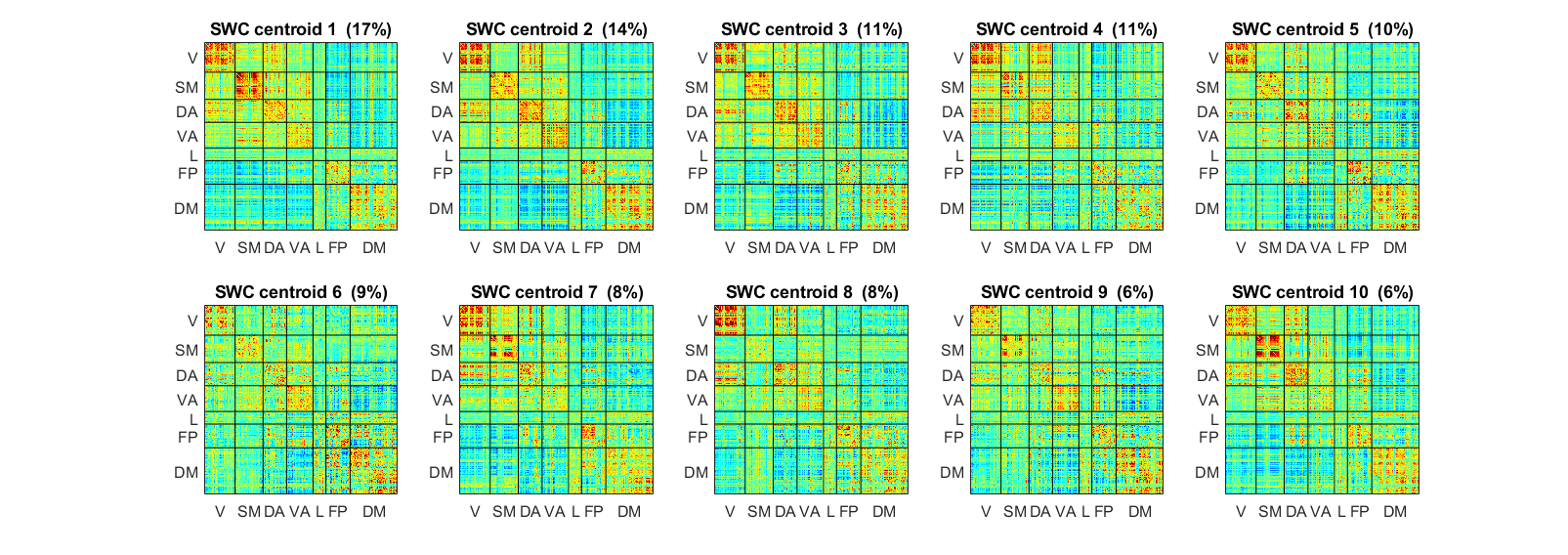
**Figure S7** Cluster centers with k=10 clusters for a subset of the data (N=20); colored by correlation (z) of parcel-pairs compared across dynamic methods. The clusters are sorted by occurrence rate (% shown above each centroid). The 360 spatial parcels are sorted into 7 major functional networks identified by Yeo et al. (Yeo, Krienen et al. 2014).


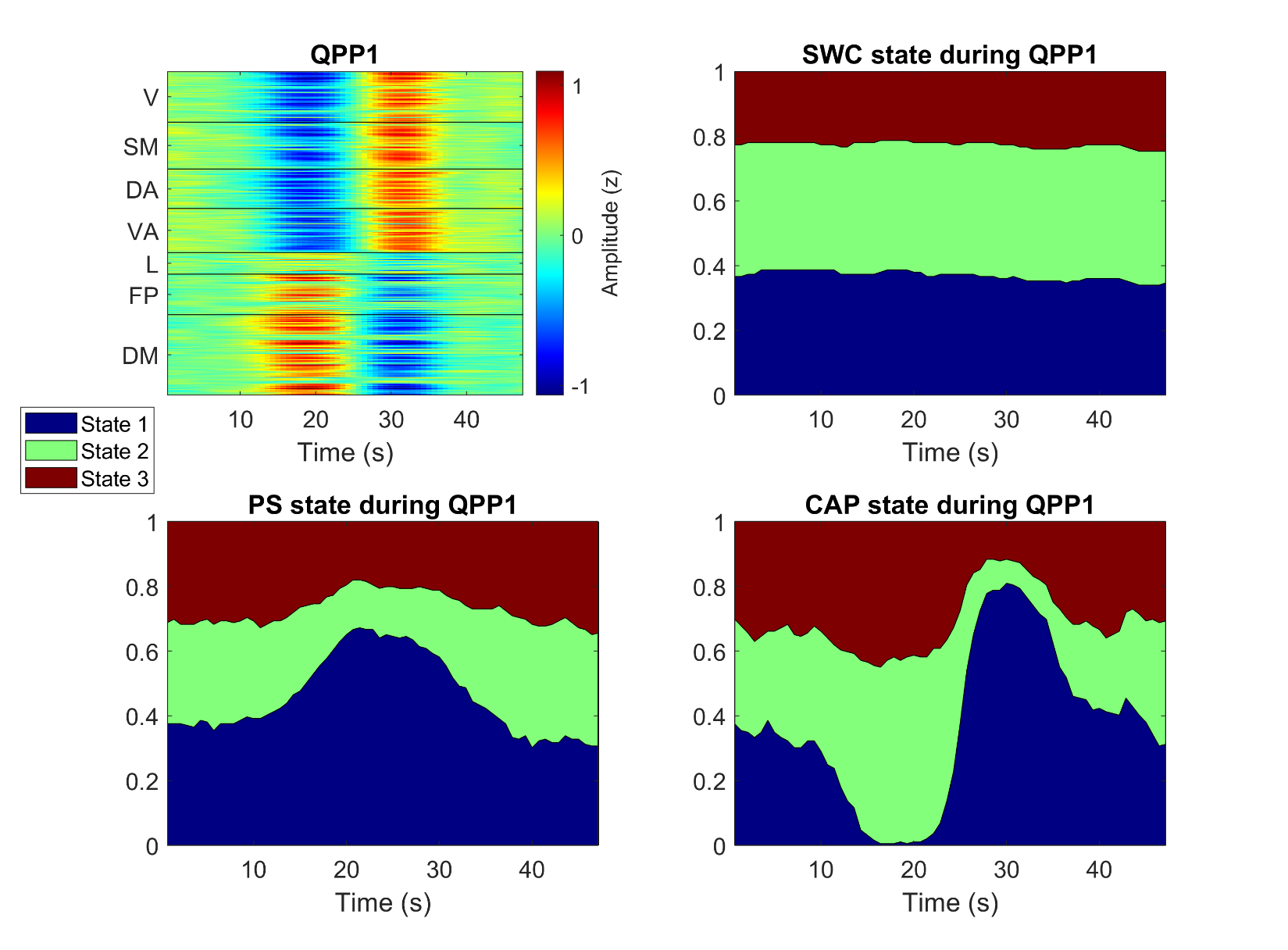


**Figure S8** Comparison of most likely states during QPP1 with k=3 clusters for a subset of the data (N=20). Top-left shows spatiotemporal QPP1 template with timepoints on the x-axis and space (Glasser’s 360 parcels) on the y-axis and colors corresponding to z-score BOLD signal amplitude. The other 3 panels show the same timepoints on the x-axis with the likelihood of being in each state plotted on the y-axis with the colors corresponding to the 3 brain states identified by each dynamic analysis method.


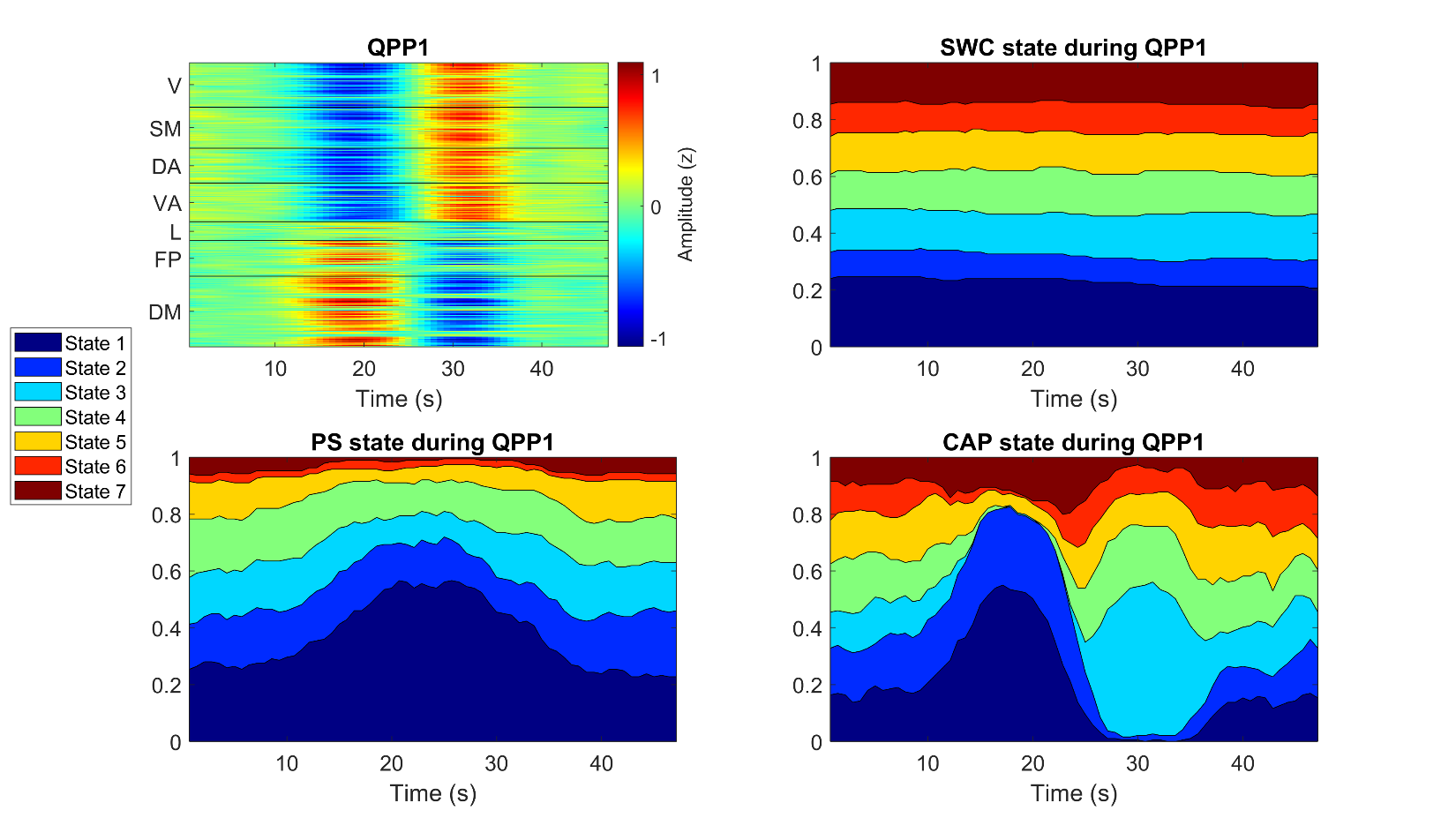


**Figure S9** Comparison of most likely states during QPP1 with k=7 clusters for a subset of the data (N=20). Top-left shows spatiotemporal QPP1 template with timepoints on the x-axis and space (Glasser’s 360 parcels) on the y-axis and colors corresponding to z-score BOLD signal amplitude. The other 3 panels show the same timepoints on the x-axis with the likelihood of being in each state plotted on the y-axis with the colors corresponding to the 7 brain states identified by each dynamic analysis method.


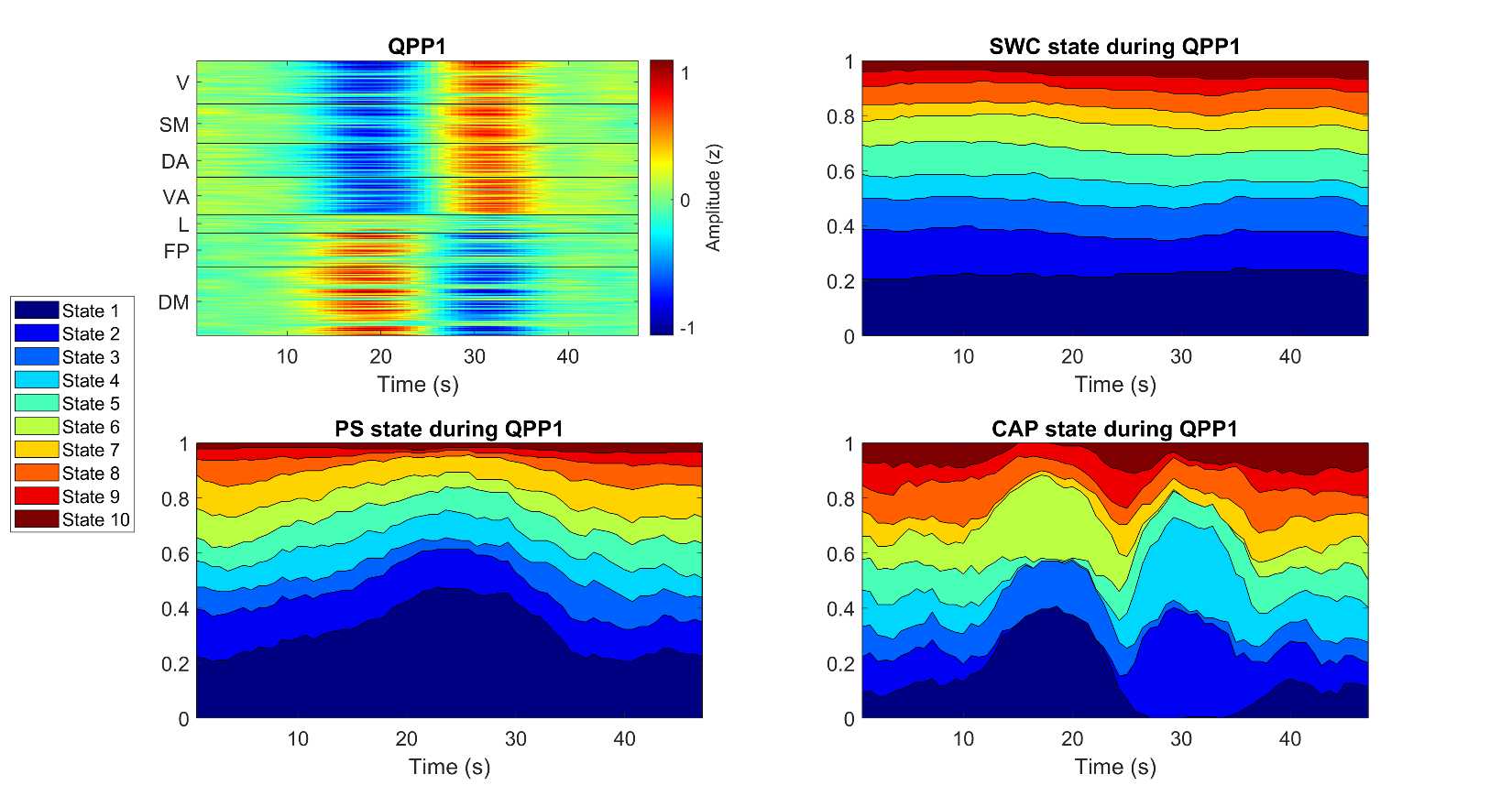


**Figure S10** Comparison of most likely states during QPP1 with k=10 clusters for a subset of the data (N=20). Top-left shows spatiotemporal QPP1 template with timepoints on the x-axis and space (Glasser’s 360 parcels) on the y-axis and colors corresponding to z-score BOLD signal amplitude. The other 3 panels show the same timepoints on the x-axis with the likelihood of being in each state plotted on the y-axis with the colors corresponding to the 10 brain states identified by each dynamic analysis method.


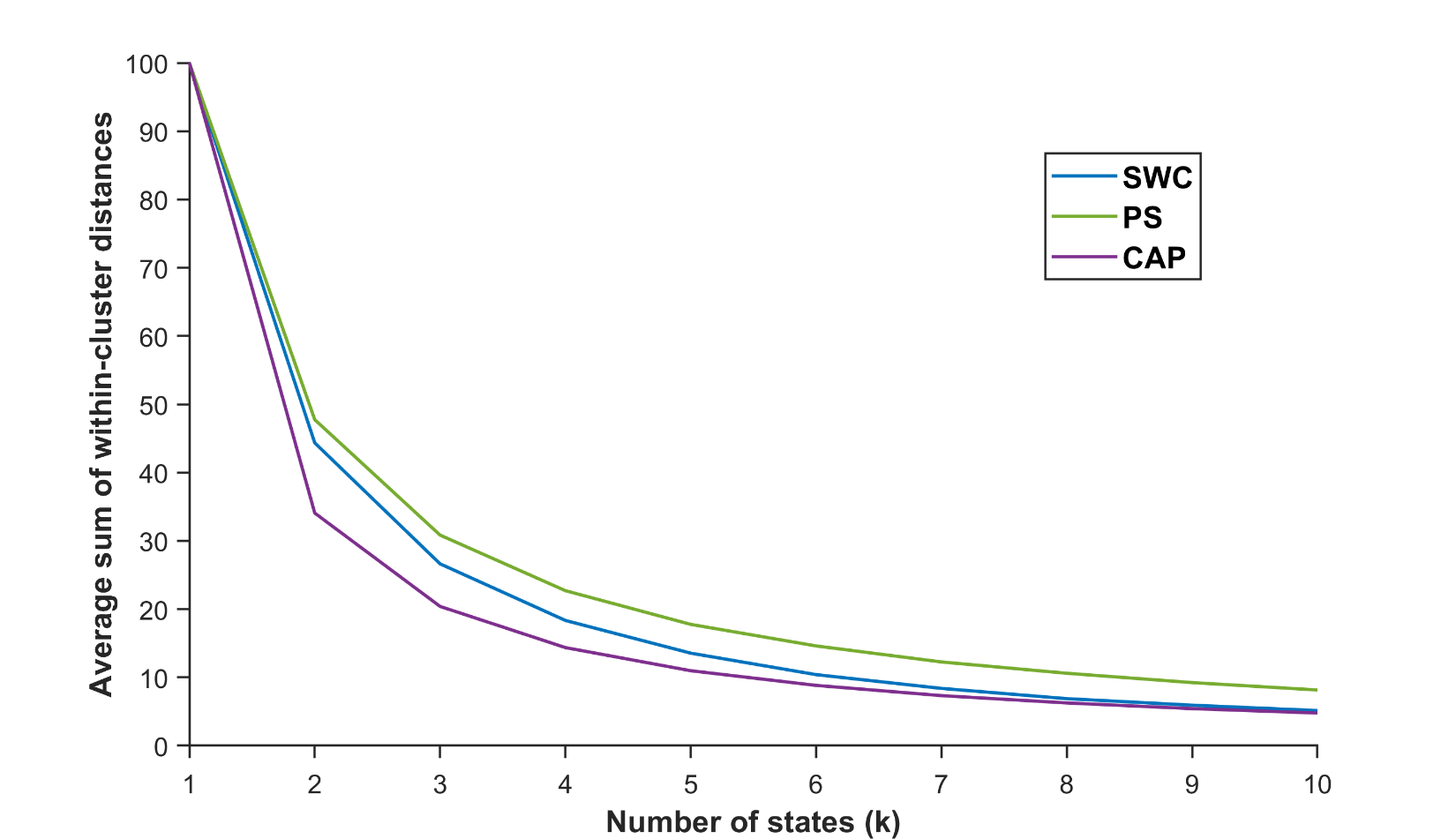


**Figure S11 –** Plot of the average sum of within-cluster distances as a percentage of the total sum of distances in the data for each analysis method over a range of k = 2:10 clusters.
